# Harnessing interactive technologies to improve health outcomes in juvenile idiopathic arthritis

**DOI:** 10.1186/s12969-017-0168-y

**Published:** 2017-05-16

**Authors:** Andrea Coda, Dean Sculley, Derek Santos, Xavier Girones, Lucie Brosseau, Derek R. Smith, Joshua Burns, Keith Rome, Jane Munro, Davinder Singh-Grewal

**Affiliations:** 1 School of Health Sciences, Faculty of Health and Medicine, The University of Newcastle, Ourimbah, Australia; 2 School of Biomedical Sciences and Pharmacy, Faculty of Health and Medicine, The University of Newcastle, Ourimbah, Australia; 3 School of Health Sciences, Queen Margaret University, Edinburgh, UK; 4 Faculty of Health Sciences at Manresa, University of Vic-Central University of Catalonia, Manresa, Barcelona Spain; 5 School of Rehabilitation, Ottawa University, Ottawa, Canada; 6 James Cook University, Townsville, Australia; 7 The Children’s Hospital at Westmead & the University of Sydney, Hawkesbury Rd & Hainsworth St, Sydney, NSW 2000 Australia; 8 AUT University, Auckland, New Zealand; 9 Department of General Medicine, Royal Children’s Hospital, Parkville, VIC Australia; 10 Sydney Children Hospitals Network & Clinical A/Prof- The University of Sydney, Sydney, Australia

## Abstract

**Background:**

Children and adolescents with Juvenile Idiopathic Arthritis (JIA) typically have reduced physical activity level and impaired aerobic and anaerobic exercise capacity when compared to their non-JIA counterparts. Low intensity exercise regimens appear to be safe in children with JIA and may results in improvements in overall physical function. Poor adherence to paediatric rheumatology treatment may lead to negative clinical outcomes and possibly increased disease activity. This includes symptoms such as pain, fatigue, quality of life, longer term outcomes including joint damage, as well as increase of healthcare associated costs. Low adherence to medications such as methotrexate and biological-drugs remains a significant issue for paediatric rheumatologists, with alarming reports that less than half of the children with JIA are compliant to drug-therapy.

**Main body:**

The recent advances in interactive technology resulting in a variety of wearable user-friendly smart devices may become a key solution to address important questions in JIA clinical management. Fully understanding the impact that arthritis and treatment complications have upon individual children and their families has long been a challenge for clinicians. Modern interactive technologies can be customised and accessed directly in the hands or wrists of children with JIA. These secured networks could be accessible ‘live’ at anytime and anywhere by the child, parents and clinicians.

Multidisciplinary teams in paediatric rheumatology may benefit from adopting these technologies to better understand domains such as patient biological parameters, symptoms progression, adherence to drug-therapy, quality of life, and participation in physical activities. Most importantly the use of smart devices technologies may also facilitate more timely clinical decisions, improve self-management and parents awareness in the progression of their child’s disease. Paediatric rheumatology research could also benefit from the use of these smart devices, as they would allow real-time access to meaningful data to thoroughly understand the disease-patterns of JIA, such as pain and physical activity outcomes. Data collection that typically occurs once every 1 or 3 months in the clinical setting could instead be gathered every week, day, minute or virtually live online. Arguably, few limitations in wearing such interactive technologies still exist and require further developments.

**Conclusion:**

Finally, by embracing and adapting these new and now highly accessible interactive technologies, clinical management and research in paediatric rheumatology may be greatly advanced.

## Background

Juvenile Idiopathic Arthritis (JIA) incurs a significant burden to patients, their families, and the community [1]. Children with JIA may experience significant issues with school attendance that has a direct impact on their academic performance [2, 3]. Hospitalisation rates in Australia for juvenile arthritis have increased from 14 to 32 per 100,000 population over the past decade [1]. A sharp 118% rise in allied health professional (AHP) involvement has also been recorded in Australia since 2005, including specialist nurses, occupational therapists, physiotherapists, and podiatrists [1]. Some of the prescribed not invasive interventions from AHP, such as regular physical exercises, could be increasingly self-managed by the child and their families and carried out without the need of attending clinics and hospitals, which may positively impact the child’s school attendance. Thus, we feel that recent advances in technology resulting in a variety of wearable user-friendly smart devices, capable of providing customised notifications, may become a key instrument in JIA clinical management. Furthermore, data collection and incorporating patient reported outcomes through interactive technologies, may play a significant role in health promotion and self-management of disease into the future [4].

### Monitoring symptoms and disease progression using interactive technologies

Understanding the impact that chronic disease and treatment complications have upon children and their families has long been a challenge for clinicians. Typically, practitioners are unable to undertake frequent assessment and so most measures, such as quality of life and disease progression, rely on recall during appointments.

A number of successful attempts have already been made in introducing smart devices to better understand patients’ disease, particularly in paediatric oncology, chronic pain, and allergic disease [5, 6]. Cingi et al. (2015), investigated 327 patients diagnosed with mild-to-severe persistent asthma and persistent allergic rhinitis (AR) that were randomised into 2 intervention groups and 2 control groups using a smart mobile platform called POPET. The intervention groups (POPET-AR and POPET-Asthma) received a mobile phone application (“physician on call patient engagement trial” [POPET]), enabling them to interact with their clinicians, and independently record their health status and compliance to their prescribed drugs. Those using the online platform had better health outcomes and quality of life, with a reduction in hospital admissions and number of medical consultations required. Furthermore, improvements were also recorded with regards to activity, productivity, disease perception, and emotion status. Stinson et al. (2015) investigated the validity, reliability, and feasibility of a smartphone pain assessment app among 106 children and adolescent diagnosed with cancer, and concluded that the app had excellent internal consistency, feasibility, and an overall high acceptability amongst patients.

Children and young people with JIA require active involvement in their own health decision-making and lifestyle management [7]. Pilot programs in adult rheumatic disease and other chronic health conditions have already identified the use of modern technology as a possible strategy to improve self-management; however, large scale studies specifically in JIA are still pending [8–10]. The feasibility of online self-assessment system has been performed over a 3-month period using a smart-phone application in only 9 Japanese Rheumatic Arthritis (RA) patients [8]. The smart app provided daily notifications and comments related to the disease activity [8]. The size of this study was very small and 89% of patients were women; however, the results indicated that the app was capable of longitudinally predict the disease activity score in RA patients and it may be an acceptable and useful tool for both patients and healthcare providers [8].

Jongh et al. (2012) published a Cochrane systematic review including four randomised controlled trials, involving 182 participants diagnosed with diabetes, hypertension and asthma. The studies investigated whether mobile phone App, including SMS and MMS, were able to improve self-management by providing medication reminders or supportive notifications to patients to communicate important information to their healthcare providers and exchanged prompt feedback [9]. Although the authors reported that longer studies and larger sample sizes are still necessary, overall moderate quality evidence was found suggesting positive impacts on the health of patients [9].

Majeed-Ariss et al. (2015) conducted a Systematic Review of Adolescents’ Use of Mobile Phone and Tablet Apps that Support Personal Management with their Chronic or Long-Term Physical Conditions. It included 4 papers and 46 participants with type 1 diabetes, asthma, and cancer. The authors concluded that smart devices represent a feasible health intervention; however, larger studies are still required in order to determine the Apps’ acceptability and effectiveness [10].

### Adherence to prescribed medications

Adherence to the prescribed treatment is essential so that children with JIA do not continue to endure joint symptoms, fatigue, reduced physical activity level and lower quality of life [11] and also to avoid rise of healthcare costs through increased frequency of consultations and diagnostic tests [1].

Adherence to medications including methotrexate and biological-drugs remains a significant issue in paediatric rheumatology, with alarming reports that as few as 53% of children with rheumatic disease (RD) have good overall drug-adherence [12]. Bugni et al. (2012) reported that 20 children with RD, equivalent to 20.2% of the participant recruited, had poor medication adherence related to the use of three or more daily medications (*p* = 0.047). Specifically to children with JIA, 28(51%) patients were not adherent to the prescribed medication [12]. Pelajo et al. (2012) investigated 76 outpatients and the overall rate of non-adherence to methotrexate was 18% amongst American and Portuguese children with JIA, between 1 and 17 years old. The rate of reported non-adherence was 8% in Boston and 24% in Rio de Janeiro (*P* = 0.012) [13]. Interestingly, the main reason for non-adherence in Boston was “child refused”, while in Rio de Janeiro non-adherence was mostly related to an inability to acquire the medication [13]. Multiple reasons should be considered when addressing medication adherence, such as child refusal, adverse effects or lack of medication availability, personal issues, and financial hardship [12]. Another major reason for adherence failure is forgetfulness [12]; therefore, in order to encourage children to take their prescribed drugs, parents/carers are often essential to ensuring adherence. It is possible that smart reminders, accessible directly from the patient’s mobile or tablet, may assist medication adherence.

Research in adolescents with asthma has suggested that interactive technologies should be explored as a means of improving medication adherence [14] including customised reminders accessible through a smartphone app and online information [15]. A similar approach, to test medication adherence, was successfully introduced by Crosby et al. [16] in 43 children and adolescents (age range 6–21) with sickle cell disease (SCD) showing 90% satisfaction with patients. This data highlights the potential to integrate smart web-based technology into paediatric clinical care.

### Encouraging physical activity

Children with JIA typically have reduced physical activity levels when compared to their non-JIA counterparts [11]. Poor aerobic capacity and low activity detected in children and adolescents with JIA represent a significant clinical concern, as it is likely that the reduced fitness levels will then impact their adulthood [11, 17]. Physical activity is safe in JIA and may results in improvements in physical function [18]. Thus encouraging physical activity in children with JIA is likely to have a beneficial effect. In an age where exercise in children is falling [19], the paediatric rheumatology multidisciplinary team should explore new interactive technologies to increase physical activity adherence.

Modern interactive technologies can be individualised and accessed directly in the hands or on the wrists of children with JIA. Information gathered by smart-watches (or wristbands) that are worn throughout the day by the child, already has the capacity to be wirelessly connected via Cloud networks to databases that collate physical activity data for individual and group analysis. These secured networks could be accessible ‘live’ at anytime and anywhere by the child, parents and clinicians and allow multidisciplinary teams to better understand domains such as physical activity and energy expenditure. This technology has the potential to facilitate more timely clinical decisions; improve self-management and parent’s awareness in the progression of their child’s exercise level and physical activity targets.

Smart devices can provide real-time biofeedback; the inbuilt accelerometers and heart rate monitors can measure physical activity intensity, while GPS can be employed to precisely monitor distance of movement throughout the day. As soon as the daily, weekly and/or monthly activity target has been achieved, the child with JIA could be informed (for example with a gentle vibration on the wrist) and recorded for their achievements through messages or ‘awards icons’ directly on their watch screen. These ‘award icons’ may serve as incentive and positive reinforcement for participating in a customised management plan, with the ultimate goal of encouraging the child with JIA to be more active and healthier. Future research should investigate the long-term impact of the positive reinforcements and notifications delivered by the smart devices, and possibly develop new methods to promote and sustain long term effectiveness of the interactive technologies.

### Access to interactive technologies

Today’s children and young adults already have accessible technology integrated into many aspects of their daily life [20]. They are usually comfortable with smart devices, which may engage and motivate them more effectively than traditional methods such as paper-diaries or checklists.

The rapid growth in personal technology such as tablets, smart-phones, and so on, is clearly reflected in daily usage amongst children and adolescents. In 2010, for example, 15% of teenagers in Australia owned a smartphone; and by 2013 users sharply increased to 69% of this population [20]. As smartphones are becoming more accessible and affordable, the number of young consumers is expected to continue growing such that in Australia most adolescents and many younger children are likely to have access to and use smart devices on a regular basis in the future.

### Potential new approach to paediatric rheumatology management

**Fig. 1:**
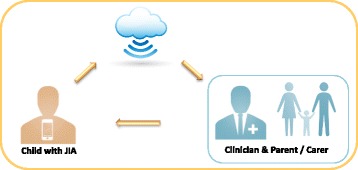
Data is automatically synchronised to the Cloud Network and instantly available remotely by the multidisciplinary JIA team and their parents/carer

With the introduction of already existing interactive technologies as part of the routine paediatric rheumatology care, we feel that new safe and cost effective approaches to care may be possible. Clinicians could connect with the child or family members by providing discreet reminders to inform that a medication is due or if symptom reporting is required. With a simple ‘tap’ on the smart-watch (or wrist band), the child could confirm when the drug was administered. Similarly, pain levels, physical activity and quality of life data may be recorded weekly, daily or even in real time. Data could be transferred to a secure ‘Cloud Platform’ for analysis and accessed by patients, their families and clinicians as appropriate. The child with JIA may benefit also from receiving personalised notifications such as text messages, from the clinicians with the aim of promoting positive behavioural changes and encourage daily self-management. The data collected may also facilitate the child, their family and the clinicians to become more aware of disease patterns (Fig. 1).

Data collection that currently occurs once every 1 or 3 months in the clinical setting could instead be gathered constantly and effortlessly, requiring minimal patient and clinician input. In addition to the ‘Cloud Platform’, data could also be used to develop logarithms to predict poor outcomes and allow immediate intervention; or foresee early flares of disease and instigate prompt interventions to minimise disruption to the patient’s daily life. This new interactive and user-focussed approach has the potential to significantly increase both the child’s and family’s engagement in their care. The introduction of smart devices to paediatric rheumatology care may provide meaningful improvements in clinical outcomes, patient well-being and engagement, with possible cost reductions for the health care system.

### Data protection and limitations

While protecting patient privacy and confidentiality of data remains paramount, many appropriate security protocols already exist amongst the major smart device providers, such as Google™ [21] and Apple™ [22] to ensure constant system security, encryption and data protection, data backup and privacy control, locations services, passwords authentication and intrusion detection. Privacy remains a primary concern for patients, parents and clinicians, especially when it is unclear where the central warehousing of data is installed, who owns the data, and who is responsible for the cost associated with these technologies.

Arguably, there are still challenges in utilising these interactive technologies, such as: the need of evidence regarding feasibility and validity of the apps being utilised and the need for regular software updates by service providers or hospital IT departments, that may impact the reliably of synchronised data transfer. In addition, missing physical activity data may occur during sports such as swimming, when wearing smart watches and wristbands might damage the device. More evidence is required to establish the willingness of children with JIA in interacting with health-related technologies especially at younger age, where children may still exhibit reading difficulties. Finally, these smart technologies might generate few unintended consequences such as increased anxiety, data hyper-vigilance and obsessive tendency in outcome such as physical activity; which need to be monitored by healthcare professionals and family members.

## Conclusion

Clearly further studies in paediatric rheumatology are required to critically evaluate the effectiveness and acceptability of available apps in large number of patients and to develop effective apps utilizing valuable input and feedback from patients, carers and clinicians. Overall, by embracing and adapting these new and now highly accessible interactive technologies, clinical management and research progress in paediatric rheumatology could be greatly advanced.
